# The Science System at the Limit: A Call to Action for Considerate Publishing

**DOI:** 10.1177/00220345251349804

**Published:** 2025-07-17

**Authors:** F. Schwendicke, S.E. Uribe, N.S. Jakubovics

**Affiliations:** 1 Clinic for Conservative Dentistry and Periodontology, LMU Klinikum Munich, Germany; 2 Department of Conservative Dentistry and Oral Health, Riga Stradins University, Riga, Latvia; 3 School of Dental Sciences, Faculty of Medical Sciences, Newcastle University, Framlington Place, Newcastle upon Tyne, UK

**Keywords:** editorial policies, open access publishing, peer review, research, science, big data

## Abstract

In recent years, the landscape of scientific publishing has experienced exponential growth, driven in part by the increasing availability of data and advanced analytical methods, by incentives set by the scientific career system and by increasing options of publication routes, with diverging rigor in peer review. While this surge offers unprecedented opportunities for exploration and innovation, it also introduces challenges that potentially compromise the quality and accessibility of scientific literature, as the number of published articles significantly outpaces the number of scientists and, hence, available peer reviewers. Moreover, the increasing complexity of scientific outputs challenges the options for comprehensive, in-depth review and reproduction. We here examine the drivers of this phenomenon, its implications for the dental research community, and potential solutions to ensure a sustainable and rigorous publishing ecosystem. Emphasizing the importance of quality over quantity, we advocate for systemic changes in academic incentives, promoting open science, and enforcing robust peer-review standards. We further summarize the recent statement of the International Committee of Medical Journal Editors toward predatory journals; the *Journal of Dental Research* actively endorses this statement.

## Introduction

The volume of scientific publications has grown exponentially for decades, increasing by approximately 5.08% each year since 1952 (Bornmann et al. 2021). There is no sign of this trend slowing; in fact, an increase in the rate of publication has been reported (Hanson et al. 2024). This growth is fostered by the availability of large-scale datasets, openly available analytic tools, and open-access journals, all of which democratize access to research and allow broader participation. In parallel, incentives set by the scientific career system (“publish or perish,” with publications being both an academic and a clinical requirement in many countries) and an increasing number of publication routes have contributed to this surge in publications, which does not necessarily come with a similar surge in true knowledge or understanding, as outlined below. Overall, this proliferation of scientific output presents a paradox. While it theoretically enables the accumulation of more knowledge and, hence, a deeper and more comprehensive understanding of oral biology, health, health care and interventions, it also creates barriers to discerning published work’s quality, reliability, and applicability.

## Drivers

Several factors have been identified that contribute to the surge in scientific publications (Hanson et al. 2024; Laine et al. 2025): (1) The widespread availability of data and analytical methodologies has lowered the barriers to entry for researchers. (2) The incentives set by the scientific career systems, with a focus on the number of publications and the usage of the number of citations of a journal (impact factor) as a metric to assign a perceived quality label to a published article, have been found to pressure scientists to publish in higher volume than ever before, partially at the expense of the true knowledge transported by a publication (e.g., splitting up research projects into several publications to increase the measured “impact”) (Trueblood et al. 2025). (3) The advent of open-access publishing models, in which authors pay fees to make their work freely available, has further increased the accessibility of scientific outputs (Grudniewicz et al. 2019; Hanson et al. 2024). In 2019, authors, institutions, and primarily taxpayers spent approximately $910 million USD on open-access publishing, a figure that has surged to $2.5 billion in 2024 (Haustein et al. 2024). As pay per publication costs continue to rise, the academic community should reflect on whether this investment is the most effective way to support scientific progress. While open-access models have democratized knowledge dissemination, the same model has been exploited by predatory journals that prioritize profits over quality. Their growth is driven by the ease of online publishing, minimal entry barriers, and the pressure on academics to publish rapidly.

Dental research has not been immune to these trends. The abundance of publications makes it increasingly challenging for clinicians and researchers to stay informed about advances in the field. Moreover, the variability in the quality of peer review—ranging from rigorous to superficial—further complicates efforts to identify reliable evidence. As discussed previously, datasets used in studies as well as the full analytical pipeline are oftentimes unavailable to peer reviewers. In addition, finding reviewers with skills and time available to appraise such studies is challenging. This issue is exacerbated by journals with streamlined editorial processes that prioritize rapid publication over thorough evaluation. As a consequence, low-quality studies often find their way into systematic reviews, where they are synthesized and inadvertently acquire the status of high-quality evidence, potentially influencing clinical guidelines and decision making (Wilkinson et al. 2025). Importantly, it is the responsibility of authors of systematic review to assess and exclude low-quality studies.

## Challenges in Maintaining Integrity

The rapid expansion of the publication landscape poses significant risks to the integrity of scientific research. In dentistry, where evidence-based practice is critical, the proliferation of low-quality studies can lead to clinical uncertainty and potentially harmful outcomes. The key challenges include the following. (1) Difficulty in knowledge appraisal and synthesis: The sheer volume of publications makes it increasingly difficult for researchers and practitioners to synthesize and critically evaluate existing evidence. This can lead to a fragmented understanding and reduced confidence in the scientific literature, by both researchers and the general public. (2) Strain on peer review: Peer review remains the cornerstone of scientific quality assurance, yet increasing submission rates have stretched the capacity of reviewers. Inconsistent review standards across journals further undermine the reliability of published work (Aczel et al. 2025). (3) Rise of predatory journals: Predatory journals exploit the author-pays model by prioritizing profit over scientific rigor. These outlets often lack robust peer review and editorial oversight, making them a significant threat to the credibility of dental research (Grudniewicz et al. 2019; Laine et al. 2025). (4) Misaligned incentives: Academic reward systems that prioritize publication quantity and related metrics over quality perpetuate a cycle of excessive and often redundant publishing, as outlined by Trueblood et al. (2025).

## Implications and Possible Solutions

For the dental research community, the strain on scientific publishing has tangible consequences. First, the inability to discern high-quality research from noise can compromise clinical guidelines, leading to suboptimal patient care. Second, early-career researchers may find it increasingly challenging to establish themselves in an environment where quantity is often valued over substantive contributions. Third, flawed studies may be used to actively inform and drive policy making; the alleged toxicity of fluoride has been cited in campaigns to ban water fluoridation, potentially increasing oral health disparities. Finally, high-profile retractions and the dissemination of poorly vetted studies can erode trust in the field, undermining its credibility and impact (Van Noorden 2025).

Addressing these challenges requires a multifaceted approach that involves systemic changes in academic culture, enhancements to the publishing process, and increased awareness among researchers. We outline several strategies to mitigate the strain on scientific publishing and its impact on dental research.

First, reforming academic incentives seems of foremost relevance. A fundamental shift in how academic success is measured is necessary to prioritize quality over quantity. Initiatives like narrative CVs, which emphasize the impact and significance of an individual’s research contributions, offer a promising alternative to traditional metrics such as publication counts. The adoption of a narrative CV or “Résumé for Researchers” by the Royal Society and UK Research and Innovation is a notable example that could inspire similar reforms in dentistry. Academia should also incentivize open science practices, such as preregistration of studies, data sharing, and adherence to the FAIR principles. By shifting the researcher’s evaluation from the quantity of publications to the transparency and reproducibility of research, the reward system can be better aligned with the values of scientific integrity and societal impact. By focusing on the real-world impact of research, these systems encourage meaningful contributions rather than superficial outputs. Similarly, academic recruitment should move beyond metrics. Committees must evaluate candidates based on qualitative assessments of their impact and scientific integrity in addition to quantitatively assessable factors. Second, peer review must be fortified to ensure the publication of high-quality research (Schwendicke et al. 2022). Journals might incentivize reviewers through recognition, awards, editorial roles, and reduced publication fees. As early-career researchers often lack training in peer review, additional training resources and processes should be considered. Collaborative peer review platforms that share the burden across institutions could also alleviate strain and improve consistency. Third, the adoption of open science practices and the strict adherence to reporting guidelines can enhance transparency and confidence in published research and facilitate replication and verification. Preregistration of study designs, the deposition of raw data, and the publication of peer review comments (open peer review) are steps toward greater accountability. Notably, intellectual property considerations and limited acceptance of open peer review by reviewers have been arguments against such practices. Fourth, efforts to identify and expose predatory journals must be intensified. Resources such as curated lists of credible journals and educational campaigns can help researchers avoid these outlets. For instance, Finland’s Publication Forum (JUFO) recently downgraded 271 journals (JUFO Publization Forum 2024). Consequently, publications in these journals will no longer be considered for hiring or promotion decisions in Finland, which sets a precedent for other academic systems to strengthen research evaluation based on quality and integrity rather than publication volume.

The dental research community should also advocate for stricter enforcement of ethical publishing standards, drawing on frameworks such as the International Committee of Medical Journal Editors (ICMJE) toward predatory journals (Laine et al. 2025). The ICMJE specifically recommends the following: (1) Authors should remain vigilant in identifying these entities: evaluating journals carefully; consulting experienced mentors, colleagues, or librarians; and using resources such as the World Association of Medical Editors’ guidelines, ThinkCheckSubmit.org, and recommendations from the U.S. National Institutes of Health (National Institutes of Health 2017) can help in this task. Authors should also verify email addresses, URLs, and other details associated with communications with journals, even contacting legitimate journals if needed to confirm authenticity. (2) Academic institutions and funding bodies should provide educational resources, such as training materials, to guide researchers, particularly early-career academics, on distinguishing legitimate journals. Librarians, with their knowledge of reliable publication venues, are essential allies in this effort. (3) Journal editors and publishers are also responsible for educating authors about predatory journals and alerting their communities to entities mimicking their operations. Cease-and-desist actions, although challenging, can deter fraudulent practices. Editors should scrutinize citations from predatory sources and notify authors when concerns arise. The *Journal of Dental Research* actively endorses this statement.

## Conclusions

The strain on scientific publishing represents a critical challenge for the dental research community. While the proliferation of publications offers opportunities for innovation, it also necessitates vigilance to maintain scientific integrity. By reforming academic incentives, strengthening peer review, promoting open science, and combating predatory practices, we can ensure that the dental literature remains a reliable foundation for clinical practice and future research. Collaborative efforts across institutions, publishers, and researchers are essential to fostering a sustainable and credible publishing ecosystem that benefits the entire field of dentistry.

## Author Contributions

F. Schwendicke, contributed to conception, design, data acquisition, analysis, and interpretation, drafted and critically revised the manuscript; S.E. Uribe, N.S. Jakubovics, contributed to conception, design, data acquisition, analysis, and interpretation, critically revised the manuscript. All authors gave final approval and agree to be accountable for all aspects of the work.
